# JMJ720 regulates flowering time in rice via H3K9 demethylation of *Hd1*

**DOI:** 10.1111/pbi.70206

**Published:** 2025-06-15

**Authors:** Xiufeng Li, Jiaping Zhu, Xiaojie Tian, Yingxiang Liu, Jiaming Wei, Zhipeng Hong, Xin Jin, Chunxiao Chen, Zhenyu Wang, Jiaqi Tang, Xianwei Song, Qingyun Bu

**Affiliations:** ^1^ State Key Laboratory of Black Soils Conservation and Utilization, Key Laboratory of Soybean Molecular Design Breeding, Northeast Institute of Geography and Agroecology Chinese Academy of Sciences Harbin China; ^2^ Laboratory of Advanced Breeding Technologies, Institute of Genetics and Developmental Biology Chinese Academy of Sciences Beijing China; ^3^ University of Chinese Academy of Sciences Beijing China

**Keywords:** rice, flowering, histone demethylation, JMJ720, Hd1

## Abstract

Heading date is a crucial agronomic trait that determines the adaptability of rice to specific planting regions and seasons. Although numerous heading date genes have been well characterized in rice, epigenetic regulation of flowering time remains limited. In this study, we identified JmjC (Jumonji C) domain‐containing gene *JMJ720* as a novel heading date‐regulated gene. Mutation of *JMJ720* leads to early flowering in both LD (long‐day) and SD (short‐day) conditions. The JMJ720 protein specifically functions as histone H3 lysine 9 demethylase (H3K9me2) *in vitro* and *in vivo*. Furthermore, we identified POH1 as an interacting protein of JMJ720, which recruits JMJ720 to regulate the histone methylation levels of *Hd1*, thereby negatively regulating rice flowering. Three JMJ720 haplotypes (Hap1, Hap2 and Hap3) were found in the world rice collection. Hap1 and Hap2 were predominant in *indica* and *japonica*, respectively, and both haplotypes showed a widespread distribution across China. Collectively, this study reveals the molecular network of epigenetic modifications involved in heading date regulation and provides new target genes for molecular breeding in rice.

## Introduction

Flowering time is a key adaptive trait that allows plants to synchronize reproduction with the most favorable environmental conditions. Rice, a typical short‐day (SD) plant, exhibits suppressed flowering under long‐day (LD) conditions. Photoperiod sensitivity plays a dominant role in controlling rice flowering. Currently, there are two conserved regulatory pathways in rice: the *Hd1‐Hd3a/RFT1* and the (*Hd1/Ghd7/DTH8*)*‐Ehd1‐Hd3a/RFT1*. The photoperiodic flowering pathway in rice has been well characterized, with key regulatory nodes Hd1 and Ehd1 controlling florigen gene expression (Hori *et al*., 2016).

*Heading date 1* (*Hd1*), the rice homologue of *CO*, plays a dual role, which suppresses flowering under LD conditions but promotes flowering under SD conditions (Yano *et al*., 2000). Hd1 dynamically regulates flowering through photoperiod‐dependent protein interactions and epigenetic modifications. Under LD conditions, Hd1 forms repressive complexes with Ghd7 that directly bind *Ehd1* cis‐elements to suppress its expression (Nemoto *et al*., 2016). Hd1 also directly interacts with DTH8 to enhance H3K27me3 deposition at the *Hd3a* locus (Du *et al*., 2017). In contrast, under SD conditions, Hd1 competes with these repressors to promote *Hd3a* and *RFT1* expression, and *Hd1* overexpression inhibits heading under both LD and SD conditions in some genetic backgrounds (Ishikawa *et al*., 2011; Zong *et al*., 2021). Additionally, Hd1 stability depends on E3 ligase HAF1 (heading date‐associated factor 1) and the vacuole autophagy pathway components ATG (Hu *et al*., 2022; Yang *et al*., 2015). Hd1 can be phosphorylated by OsK4, which might impact Hd1 stability (Sun *et al*., 2016). OsSFL1 mediated histone deacetylation on *Hd1* to dampen the expression of *Hd3a* under SD (Geng *et al*., 2020). Novel post‐translational modification regulators of *Hd1* still need to be characterized.

The JmjC (Jumonji C) domain‐containing genes encode a family of histone lysine demethylases, which are critical regulators of chromatin structure and gene expression (Chen *et al*., 2011). These enzymes catalyse the removal of methyl groups from lysine residues on histone tails, thereby modulating epigenetic marks that influence transcriptional activity (Klose *et al*., 2006). The JmjC domain is a conserved structural feature that enables these proteins to function as Fe(II) and α‐ketoglutarate‐dependent dioxygenases, which are essential for their demethylase activity (Kooistra and Helin, 2012). Phylogenetic and structural analyses of 71 JmjC proteins (20 from rice, 21 from *Arabidopsis*, 30 from human) classified them into eight evolutionarily conserved groups, demonstrating functional diversification across species (Lu *et al*., 2008).

Among the 20 JmjC proteins in rice, JMJ706 encodes a heterochromatin associated H3K9 demethylase involved in the regulation of floral organ development in rice (Sun and Zhou, 2008). Histone H3K9me2 demethylases JMJ706 and JMJ707 decrease CHG methylation at specific genomic loci during male gametogenesis in rice (Li *et al*., 2024). OsmiR396d modulates floral organ development by coordinately regulating Growth Regulating Factors (OsGRFs) and their interacting partner OsGIF1 through JMJ706 and crinkly4 receptor‐like kinase (OsCR4)‐mediated pathways (Liu *et al*., 2014a). Recent studies have shown that WUSCHEL‐related homeobox 11 (WOX11) and lateral organ boundaries domain transcription factor (LBD16) function with histone demethylase JMJ706 to control crown root development (Geng *et al*., 2024). JMJ706 promotes rice heading under SD conditions and suppresses it under LD conditions by demethylating *OsMADS51*, directly binding to *OsVIL2*, and interacting with Se14 (Yu *et al*., 2025). JMJ701/Se14 inhibits floral transition under LD conditions by demethylating H3K4me3 in the promoter region of *RFT1* (Yokoo *et al*., 2014). Flowering regulation involves not only demethylases but also numerous methyltransferases. Most SET DOMAIN GROUP (SDG) proteins have been identified, while SDG712 was the only one identified as a negative flowering regulator by increasing the H3K9me2 on *Hd3a* and *RFT1* locus (Zhang *et al*., 2021).

Developing early‐flowering rice with maintained yield facilitates climate‐resilient cropping systems through optimized growing seasons (Qiu *et al*., 2021). However, a longer growth duration does not always lead to higher grain production. To maximize rice yield, an optimal heading date gene combination must be carefully selected (Zhang *et al*., 2019b). Several genes have been identified that promote flowering without compromising yield, primarily by optimizing the balance between flowering time and resource allocation. For example, genes like *Hd1*, *Ehd1*, and *DTH8* enable early flowering by fine‐tuning responses to day length, ensuring efficient resource allocation (Sun *et al*., 2022, 2023). Florigen genes such as RFT1 and Hd3a promote early flowering while maintaining yield by synchronizing floral transition with developmental stages (Sun *et al*., 2025). Ghd7 and Ghd8 could balance vegetative and reproductive growth, so their mutations exhibit early flowering without compromising panicle development or grain filling (Wei *et al*., 2010; Yan *et al*., 2011).

Here, we identified JMJ720 (LOC_Os02g58210), a JmjC domain‐containing histone demethylase that negatively regulates rice flowering. JMJ720 interacts with the POH1 transcription factor, which promotes *Hd1* expression by recruiting JMJ720 to remove H3K9me2 marks at the *Hd1* locus. Our findings reveal a novel epigenetic regulator of flowering and elucidate its molecular mechanism, providing a potential strategy for optimizing flowering time without compromising yield.

## Results

### Isolation and functional analysis of JMJ720

**Figure 1:**
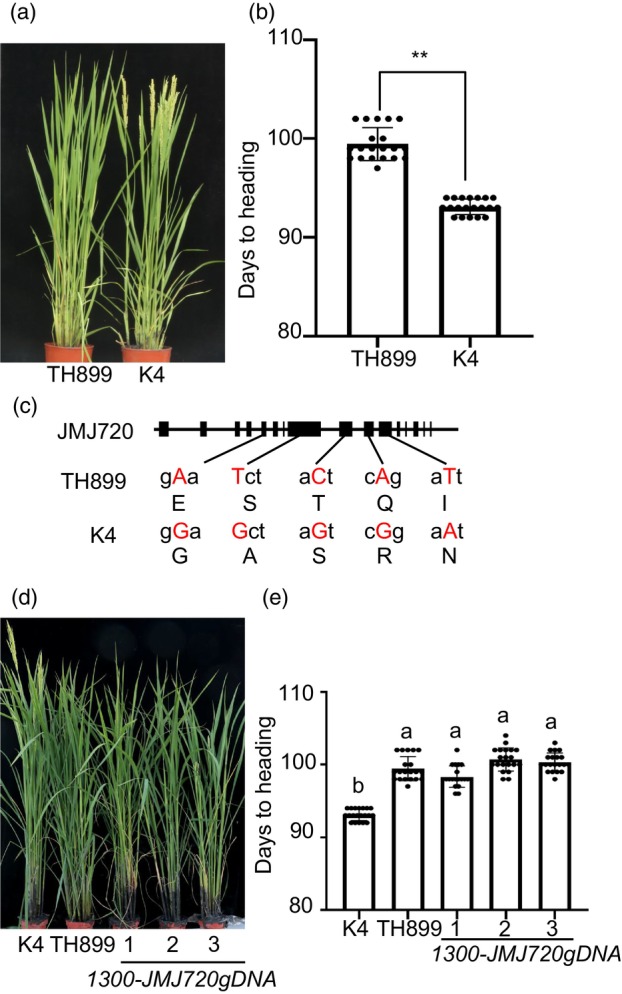
Functional analysis of JMJ720. (a) Phenotype of TH899 and K4 grown under LD conditions. (b) Heading date of TH899 and K4 under LD conditions. Data are means ± standard error (SE; *n* = 20). *P* values were calculated by Student's *t*‐test compared to TH899; **P < 0.01. (c) Sequence differences at the JMJ720 locus between TH899 and K4. (d) Phenotype of TH899, K4 and complemented plants grown under LD conditions. (e) Heading date of TH899, K4 and complemented plants under LD conditions. Data are means ± standard error (SE; *n* = 20). Statistically significant differences are indicated by different lowercase letters (*P* < 0.05, one‐way ANOVA with Tukey's significant difference test).

To identify minor heading date regulators with breeding potential in rice, we selected two major cultivars from Northeast China that exhibit subtle differences in flowering time. The K4 flowered earlier than Tonghe 899 (TH899) by around 6.3 days in natural LD condition (Figure 1a,b). TH899 and K4 were crossed to generate a genetic population, and the F_1_ progeny displayed a similar heading date to TH899. The F_2_ population segregated into early‐ and late‐heading plants in a 96:310 ratio (*χ* ^2^ = 0.397, *P* < 0.01), indicating that the flowering difference between TH899 and K4 was caused by a single Mendelian locus. We performed bulked segregant analysis (BSA) using extreme early‐flowering and late‐flowering individuals from the F_2_ population. Genome‐wide SNP/INDEL index calculations revealed a significant peak on chromosome 2, pinpointing the candidate genomic region (Figure S1a). Within the interval, we identified a 917.6‐kb genomic region containing 85 predicted genes (http://rice.uga.edu/cgi‐bin/gbrowse/rice/), and the sequences of 85 open reading frames (ORFs) were compared between TH899 and K4. Previous studies have revealed that the JmjC domain‐containing demethylases are involved in flowering in rice and *Arabidopsis* (Crevillen *et al*., 2014; Dutta *et al*., 2017; Hung *et al*., 2021; Jeong *et al*., 2009; Lu *et al*., 2010; Noh *et al*., 2004; Yang *et al*., 2012a,b; Yokoo *et al*., 2014; Yu *et al*., 2025). We then chose the *JMJ720* (LOC_Os02g58210), a member of the JMJ family, as a strong candidate gene. There were five nonsynonymous SNPs in the JMJ720 coding sequence difference between TH899 and K4. The G/A, G/T, G/C, G/A and A/T transition in the codon of *JMJ720* causes E204G, S434A, T643S, Q688R and I798N amino acid substitution (Figure 1c). To verify whether JMJ720 is the causal gene, we generated complementation plants by introducing an 8700‐bp fragment of JMJ720 from TH899 (containing both promoter and genomic sequences) into the K4 background. Three independent lines exhibiting comparable expression levels were selected for subsequent analysis (Figure S1b). Phenotypic analysis demonstrated that the complementation lines exhibited significantly delayed flowering compared to K4, with heading dates comparable to TH899 (Figure 1d,e). These results demonstrate that JMJ720 is the causal gene responsible for the heading date variation between K4 and TH899.

### JMJ720 loss‐of‐function mutant displays early flowering

**Figure 2:**
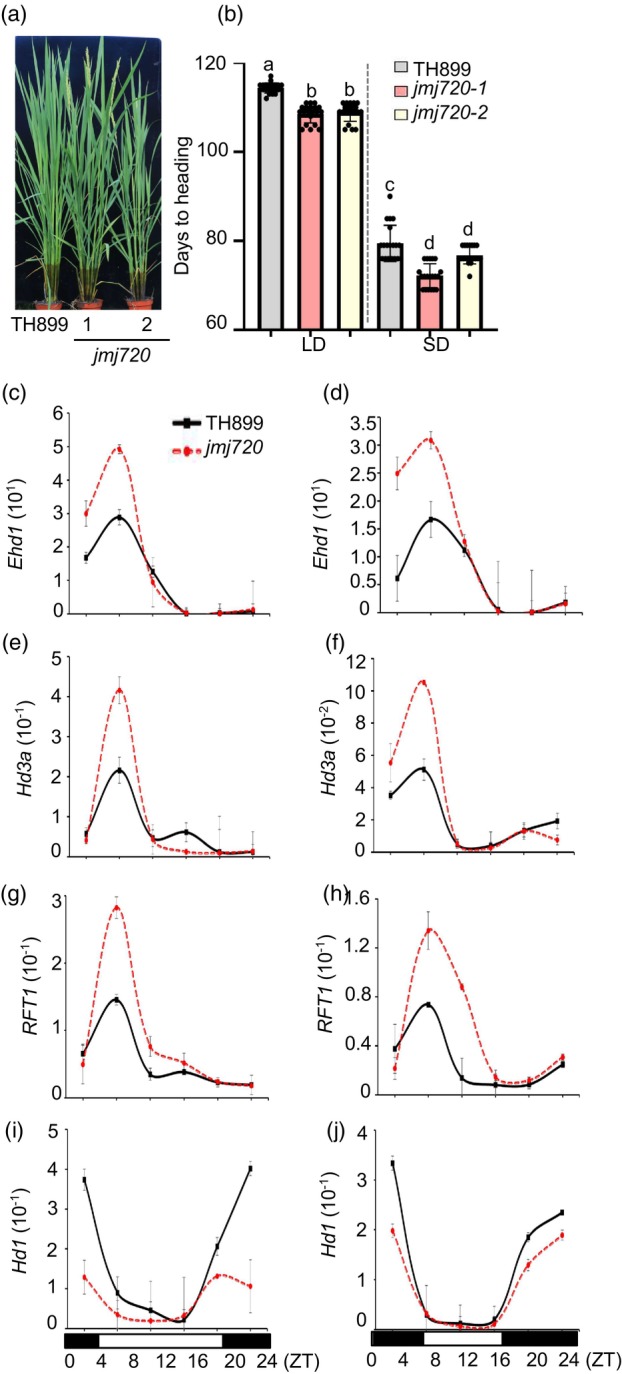
*jmj720* mutants show early flowering. (a) Phenotype of *jmj720* and TH899 plants grown under LD conditions. (b) Heading date of *jmj720* and TH899 plants under LD and SD conditions. Data are means ± SE (*n* = 20). Statistically significant differences are indicated by different lowercase letters (*P* < 0.05, one‐way ANOVA with Tukey's significant difference test). (c–j) Rhythmic expression patterns of *Ehd1* (c, d), *Hd3a* (e, f), *RFT1* (g, h) and *Hd1* (i, j) in *jmj720* and TH899 plants under LD (c, e, g, i) and SD (d, f, h, j) conditions. Black and white boxes denote dark and light periods, respectively. *UBIQUITIN* was used as the internal control. Data are means ± SE (*n* = 3). ZT, zeitgeber time.

To further investigate the function of JMJ720 involved in heading date, we generated *jmj720* knockout mutants in the TH899 background using CRISPR‐Cas9‐mediated gene editing. Two homozygous *jmj720* mutants were generated (Figure S2a,b). It was shown that *jmj720‐1* and *jmj720‐2* flowered around 5.6‐day earlier than TH899 under LD conditions (14 h light/10 h dark) and 4.9‐day earlier under SD conditions (10 h light/14 h dark) (Figure 2a,b). Remarkably, over two consecutive years of observation, *jmj720* mutants exhibited comparable agronomic traits to TH899, including plant height, panicle number, panicle length, grain number per panicle, 1000‐grain weight and grain yield per plant (Figure S3). Together, our results indicate that *jmj720* mutants have the potential to moderately promote flowering without compromising yield.

To confirm this result, we also generated another two CRISPR/Cas9 knockout lines (*jmj720‐3* and *jmj720‐4*) in *japonica* cultivar Zhonghua 11 (ZH11) background, which shares the same *JMJ720* genotype with TH899 (Figure S2c). Both *jmj720‐3* and *jmj720‐4* displayed significantly earlier heading dates than ZH11, showing 6.5‐day and 14.8‐day acceleration under SD and LD conditions, respectively (Figure S2d,e), which are similar to *jmj720* mutants in TH899 background.

To elucidate the molecular mechanism of JMJ720 in photoperiod‐mediated flowering regulation, we examined the expression of key flowering‐related genes in *jmj720* mutants. qPCR analysis showed significantly higher expression levels of *Ehd1*, *Hd3a* and *RFT1* in *jmj720* mutants compared to TH899 (Figure 2c–h). Conversely, transcript levels of flowering repressors (*Hd1*, *Hd4* and *Hd5*) were markedly reduced in *jmj720* mutants compared to TH899 under either SD or LD conditions (Figures 2i,j and S4). These observations are consistent with the early‐flowering phenotype caused by *jmj720* mutation and suggest that JMJ720 might be involved in a photoperiod‐dependent pathway.

To further characterize JMJ720 function, its CDS fused with a Flag tag was overexpressed in ZH11 (Figure S5a). The *JMJ720 OE* plants with higher levels of *JMJ720* expression were identified via an RT‐qPCR analysis and Western blot (Figure S5b,c). However, the heading dates of *JMJ720 OE* were similar to that of ZH11 under either SD or LD conditions (Figure S5d), indicating elevated *JMJ720* expression does not alter flowering phenotype.

### Expression pattern of JMJ720 and subcellular localization

**Figure 3:**
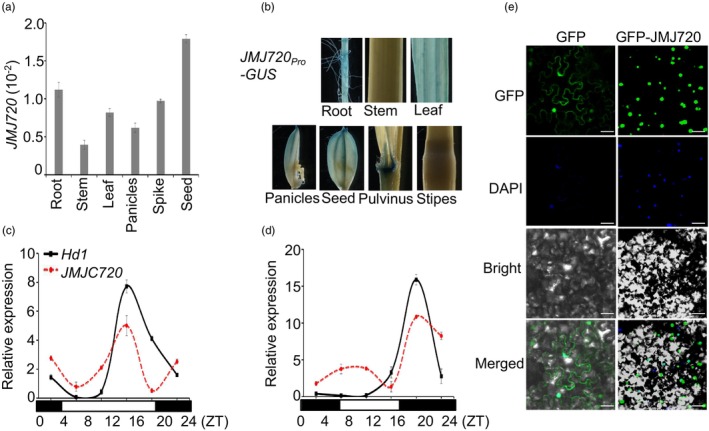
Expression pattern and subcellular localization of JMJ720. (a) Expression levels of *JMJ720* in different tissues (*n* = 3). (b) The spatial expression pattern of *JMJ720* was determined by histochemical staining of GUS activity in *JMJ720*_*Pro*_ *:GUS* plants. (c, d) Rhythmic expression patterns of *Hd1* and *JMJ720* in ZH11 plants under LD (c) and SD (d) conditions. Black and white boxes denote dark and light periods, respectively. *UBIQUITIN* was used as the internal control. Data are means ± SE (*n* = 3). ZT, zeitgeber time. (e) Subcellular localization of JMJ720 in *N. benthamiana* leaves. *Agrobacteria* harboring *35S*_*Pro*_ *:GFP‐JMJ720* was infiltrated into *N. benthamiana* and the GFP signal was observed at 48 h after infiltration. *35S*_*Pro*_ *:GFP* was used as a control. The positions of nuclei were shown by DAPI staining. Bars, 50 μm.

To characterize the spatiotemporal expression pattern of *JMJ720*, we performed RT‐qPCR analysis across multiple tissues. *JMJ720* transcripts were ubiquitously detected in roots, stems, leaves, panicles, spikes and seeds, with maximal accumulation in seeds (Figure 3a). To further examine its tissue‐specific expression patterns, we generated transgenic plants expressing a β‐glucuronidase (GUS) reporter driven by the *JMJ720* promoter (*JMJ720*_*Pro*_ *:GUS*). Histochemical GUS staining was observed in roots, leaves, panicles and seeds, demonstrating widespread expression (Figure 3b), consistent with the transcript expression pattern results (Figure 3a). To investigate the photoperiodic expression patterns of *JMJ720*, we used 40‐day ZH11 plants grown under LD and SD conditions. *JMJ720* exhibited robust circadian oscillations, displaying expression peaks at dusk under LD and during nighttime under SD conditions, exhibiting similar expression rhythms to *Hd1* (Figure 3c,d). To investigate the subcellular localization of JMJ720, we performed transient expression assays in *Nicotiana benthamiana* leaves. We observed GFP‐JMJ720 fluorescence in nuclei overlapped with 4,6‐diamidino‐2‐phenylindole (DAPI) stained regions (Figure 3e), establishing JMJ720 as a nuclear protein, supporting its predicted function as a chromatin‐modifying enzyme.

### JMJ720 specifically demethylates histone H3K9me2 *in vivo* and *in vitro*

**Figure 4:**
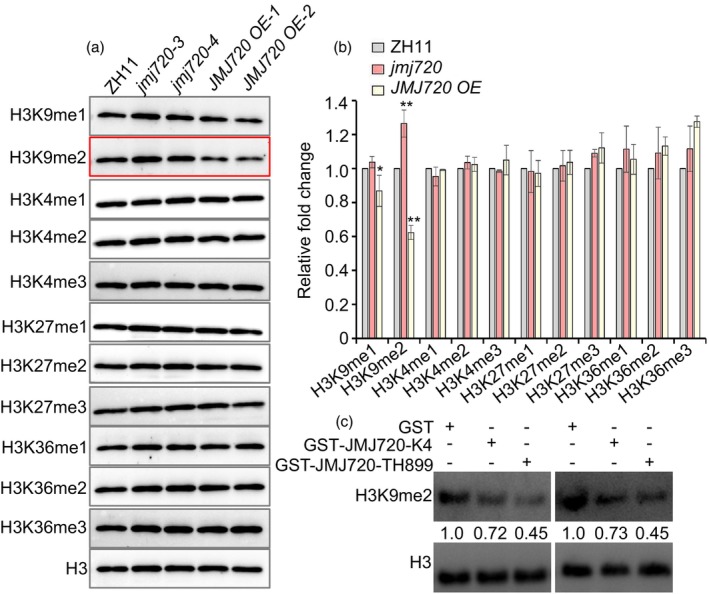
JMJ720 histone demethylation activity. (a) Global histone methylation profiles of ZH11, *jmj720‐3*, *jmj720‐4*, *JMJ720 OE‐1* and *JMJ720 OE‐2* with indicated antibodies. (b) Quantification of relative levels of H3 methylated at K9, K4, K27 or K36. Values are means ± SD of three biological. *P* values were calculated by Student's *t* test, * is *P* < 0.05 and ** is *P* < 0.01. (c) GST‐JMJ720 exhibits H3K9 demethylase activity *in vitro*. The left and right panels show the results of two independent experimental replicates, respectively.

As a Class IV JHDM2 family member, JMJ720 was predicted to possess histone demethylase activity based on sequence homology (Sun and Zhou, 2008). To assess the enzymatic specificity of JMJ720 *in vivo*, we quantified endogenous histone methylation levels using modification‐specific antibodies in nuclei isolated from ZH11, *jmj720* mutants and *JMJ720 OE* plants. Immunoblot analysis revealed significantly elevated H3K9me2 levels in *jmj720* mutants nuclei compared to WT, while *JMJ720 OE* plants showed reduced H3K9me2 accompanied by a slight decrease of H3K9me1 (Figure 4a,b). By contrast, there were no significant changes in methylation levels of H3K4, H3K27 or H3K36 among *jmj720* mutants and *JMJ720 OE* lines, demonstrating remarkable substrate specificity for H3K9. To biochemically characterize the enzymatic activity of JMJ720, we performed *in vitro* demethylation assays using prokaryotic expression proteins as described previously (Huang *et al*., 2019). GST‐JMJ720 variants (GST‐JMJ720‐TH899 and GST‐JMJ720‐K4) were purified from *Escherichia coli* cells and incubated with histone substrates. Immunoblot analysis revealed that both recombinant proteins significantly reduced H3K9me2 levels compared to GST controls. Intriguingly, GST‐JMJ720‐TH899 displayed enhanced demethylase activity relative to GST‐JMJ720‐K4, suggesting those five amino acid substitutions between TH899 and K4 indeed affect its demethylation activity (Figure 4c). These collective results establish that JMJ720 is primarily involved in the demethylation of H3K9 in rice, with lower enzymatic activity in K4 than TH899.

### JMJ720 modulates H3K9me2 levels at the *Hd1* locus

**Figure 5:**
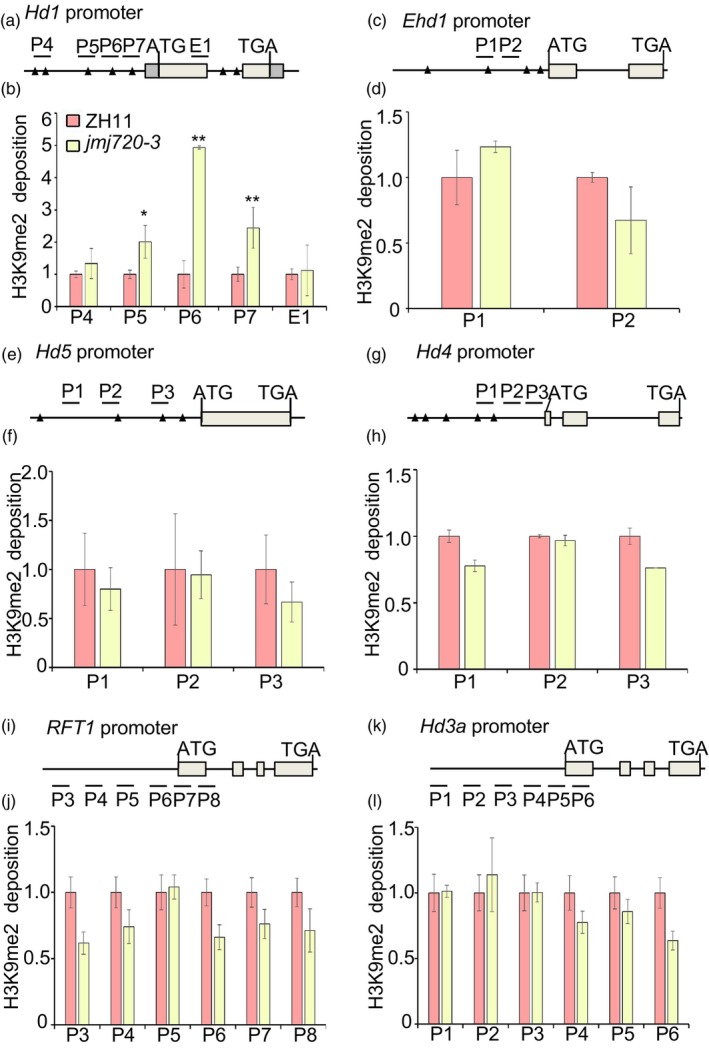
H3K9me2 enrichment at *Hd1*, *Ehd1*, *Hd4*, *Hd5*, *RFT1* and *Hd3a* locus in ZH11 and *jmj720*. The schematic diagrams of *Hd1* promoter (a), *Ehd1* promoter (c), *Hd4* promoter (e), *Hd5* promoter (g), *RFT1* promoter (i) and *Hd3a* promoter (k). The E‐box (CANNTG) containing region was indicated. Short lines indicate the region used for detection by ChIP‐qPCR. Immunoprecipitation was performed with anti‐H3K9me2 antibody in ZH11 and *jmj720*. The amount of H3K9me2 enrichment at *Hd1* (b), *Ehd1* (d), *Hd4* (f), *Hd5* (h), *RFT1* (j) and *Hd3a* (l) was quantified by qPCR. Data were shown as means ± SE (*n* = 3) relative to ZH11. *P* values were calculated by Student's *t*‐test, * is *P* < 0.05 and ** is *P* < 0.01.

Our previous results showed that the peak expression levels of *Hd1*, *Hd4* and *Hd5* were reduced in *jmj720* (Figures 2i,j and S4), while those of *Ehd1*, *Hd3a* and *RFT1* were elevated (Figure 2c–h). Given that JMJ720 functions as an H3K9me2 histone demethylase, to determine whether these expression changes resulted from direct chromatin regulation, we performed chromatin immunoprecipitation followed by quantitative PCR (ChIP‐qPCR) assays using an H3K9me2‐specific antibody in *jmj720* mutants. As shown in Figure 5, several genomic regions of each gene including the promoter and first exon were selected for qPCR analysis. We found that the H3K9me2 level was increased in the promoter region at the *Hd1* locus (Figure 5b), whereas no significant differences were observed at other tested loci between *jmj720*‐3 and ZH11 (Figure 5d–l), suggesting that JMJ720 regulates H3K9me2 demethylation of *Hd1*. To test the possibility of the interaction between JMJ720 and Hd1, we performed a series of biochemical assays. However, both yeast two‐hybrid (Y2H) and luciferase complementation imaging (LCI) systems failed to detect any physical interaction between JMJ720 and Hd1 (Figure S6a,b). Taken together, these results indicate that JMJ720 might be involved in flowering time regulation by removing the repressive marker H3K9me2 at the *Hd1* locus and do not directly regulate the expression of *Hd4*, *Hd5*, *Ehd1*, *Hd3a* and *RFT1* by modulating their H3K9me2 methylation status.

### JMJ720 physically interacts with POH1 to regulate flowering time in rice

**Figure 6:**
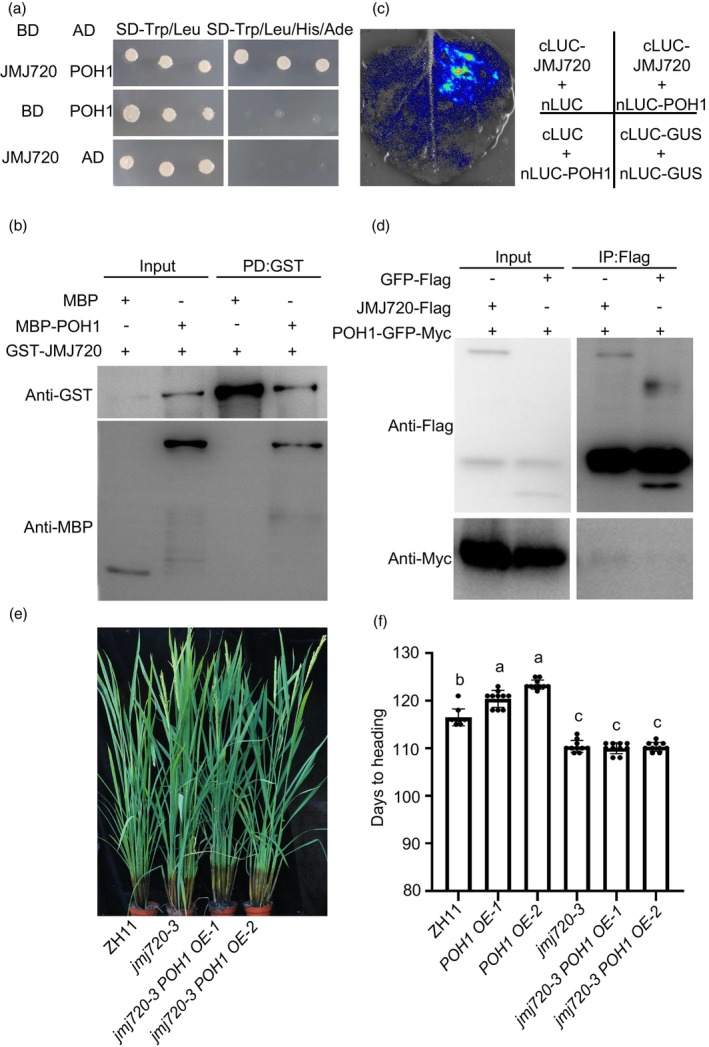
POH1 physically interacts with JMJ720 and its functionality depends on JMJ720. (a) POH1 interacts with JMJ720 in yeast cells. The indicated construct pairs were cotransformed into yeast strain Y2H. AD: pGADT 7 vector, BD: pGBKT7 vector. (b) Pull down analysis showing POH1 interacts with JMJ720 *in vitro*. (c) LUC complementation assay showing POH1 interacts with JMJ720 in *N. benthamiana* leaves. (d) Co‐IP analysis showing POH1 interacts with JMJ720 *in vivo*. (e) Phenotype of ZH11, *jmj720‐3* and *jmj720‐3 POH1 OE* grown under SD conditions (phenotype of *POH1 OE* plants and ZH11 are shown in Figure S7a). (f) Heading date of ZH11, *POH1 OE*, *jmj720‐3* and *jmj720‐3 POH1 OE* under SD conditions. Data are means ± standard error (SE; *n* = 10). Statistically significant differences are indicated by different lowercase letters (*P* < 0.05, one‐way ANOVA with Tukey's significant difference test).

As JMJ720 suppresses flowering and modulates the H3K9me2 methylation status of *Hd1*, we attempt to investigate the molecular mechanism by which JMJ720 regulates *Hd1*. Previous studies have shown that JMJ proteins interact with transcription factors to regulate the methylation status of target genes, and *Arabidopsis* JMJ28 interacts with FLOWERING BHLH (FBH) to remove repressive H3K9me2 marks at the *CO* locus (Hung *et al*., 2021). In addition, rice has two FBH homologues named Hd1 Binding Protein 1 (HBP1, LOC_Os04g41229) and Partner of HBP1 (POH1, LOC_Os08g39630), and they function as flowering repressors by activating *Hd1* expression under both LD and SD conditions (Yin *et al*., 2023). Given the similar circadian expression patterns of *JMJ720* and *Hd1* (Figure 3c,d), we investigated whether JMJ720 participates in POH1‐mediated *Hd1* regulation. Firstly, we performed yeast two‐hybrid assays and found that JMJ720 could interact with POH1 (Figure 6a), but not with HBP1. To validate the interaction, the pull‐down assay was performed. It was shown that MBP‐POH1 was pulled down by GST‐JMJ720 immobilized on Glutathione beads (Figure 6b), indicating that JMJ720 physically interacts with POH1 *in vitro*. We then examined the interaction using the LUC complementation imaging assay. The strong luminescence signal was observed when cLUC‐JMJ720 and nLUC‐POH1 were coexpressed (Figure 6c) Conversely, no LUC activity was detected from cLUC‐JMJ720 and nLUC, nLUC‐POH1 and cLUC or cLUC‐GUS and nLUC‐GUS combinations (Figure 6c). Furthermore, the Co‐IP assay was performed, and it showed that, compared with GFP‐Flag, POH1‐GFP‐Myc could be immunoprecipitated together with JMJ720‐Flag in rice protoplasts (Figure 6d), demonstrating that JMJ720 associates with POH1 *in vivo*.

As JMJ720 physically interacts with POH1, we asked whether *POH1* overexpression delayed flowering time depending on JMJ720. To test this, we generated *POH1* overexpressing (*POH1 OE*) lines in both ZH11 and *jmj720‐3* mutant backgrounds, each obtaining two independent lines with comparable expression levels (Figure S7a,b). Under SD conditions, *POH1 OE* plants flowered 5.4‐day later than ZH11 (Figures S7a and 6f), while the *jmj720‐3 POH1 OE* double mutants flowered at the same time as *jmj720‐3*, both 11.7‐day earlier than the *POH1 OE* lines (Figures S7a and 6e,f). These results suggest that JMJ720 is essential for the flowering repressive function of POH1.

### JMJ720 can be recruited by POH1 to directly target the *Hd1* locus

Previous studies have demonstrated that *Hd1* is a direct transcriptional target of POH1 (Yin *et al*., 2023). To validate the direct binding of POH1 to *Hd1*, we performed ChIP‐qPCR assays using the Myc antibody in *POH1 OE* lines. Consistent with previous findings, POH1 specifically binds to the promoter of *Hd1* and activates its expression (Yin *et al*., 2023) (Figure S8).

**Figure 7:**
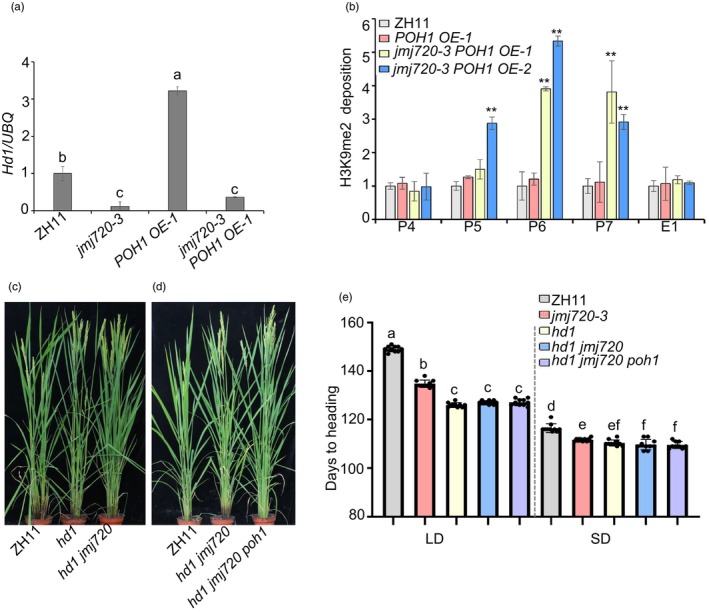
POH1 activate *Hd1* expression by recruiting JMJ720 to remove H3K9me2 from the *Hd1* locus. (a) RT‐qPCR analysis of *Hd1* transcript levels in 60 days plants grown under LD conditions. *UBIQUITIN* was used as the internal control. Statistically significant differences are indicated by different lowercase letters (*P* < 0.05, one‐way ANOVA with Tukey's significant difference test). Data are means ± SE (*n* = 3). (b) ChIP assay. Immunoprecipitation was performed with anti‐H3K9me2 antibody in *POH1 OE*, *jmj720‐3* and *jmj720‐3 POH1 OE*. The amount of H3K9me2 enrichment at *Hd1* was quantified by qPCR. Data was shown as means ± SE (*n* = 3) relative to ZH11. *P* values were calculated by Student's *t*‐test, ** is *P* < 0.01. (c, d) Phenotype of ZH11, *hd1*, *hd1 jmj720* and *hd1 jmj720 poh1* grown under LD conditions. (e) Heading date of ZH11, *hd1*, *hd1 jmj720* and *hd1 jmj720 poh1* under LD and SD conditions. Data are means ± standard error (SE; *n* = 10). Statistically significant differences are indicated by different lowercase letters (*P* < 0.05, one‐way ANOVA with Tukey's significant difference test).

To determine whether JMJ720 regulates flowering through a POH1‐*Hd1*‐dependent pathway, we first analysed *Hd1* expression across different genetic backgrounds. RT‐qPCR revealed that *Hd1* transcript levels were significantly up‐regulated in the *POH1 OE‐1* line but substantially down‐regulated in the *jmj720‐3* mutant compared to ZH11 (Figure 7a). Notably, in the *jmj720‐3 POH1 OE‐1* double mutant, the POH1‐mediated up‐regulation of *Hd1* was largely abolished, indicating that JMJ720 is essential for POH1‐dependent activation of *Hd1* expression (Figure 7a).

Given the observed reduction of *Hd1* expression in *jmj720* mutants (Figures 2i,j and 7a), we postulated that POH1 might recruit JMJ720 to modulate the H3K9me2 methylation level of *Hd1*. To test this hypothesis, we performed ChIP‐qPCR assays using an H3K9me2‐specific antibody in *POH1 OE‐1* and *jmj720‐3 POH1 OE* double mutant plants to examine the enrichment of *Hd1*. Consistent with our expectations, the H3K9me2 methylation level of *Hd1* accumulated in the *jmj720‐3 POH1 OE* double mutants but remained unchanged in *POH1 OE‐1* (Figure 7b), demonstrating that POH1 requires functional JMJ720 to maintain the H3K9me2 methylation level of *Hd1*.

To further elucidate the genetic relationship among POH1, JMJ720 and Hd1 in flowering regulation, we generated a series of mutants in the ZH11 background: *hd1*, *poh1*, *hd1 jmj720* and *hd1 jmj720 poh1* triple mutant (Figure S9a,b). These plants were cultivated under LD and SD conditions to assess their heading dates. Phenotypic analysis revealed that the *hd1* mutant exhibited earlier flowering under both LD and SD conditions, with a particularly pronounced acceleration of 23.1 days than ZH11 under LD (Figure 7c,e). The *poh1* mutants displayed flowering time comparable to ZH11, consistent with a previous report (Yin *et al*., 2023) (Figure S9c,d). However, both *hd1 jmj720* double mutant and *hd1 jmj720 poh1* triple mutant showed flowering phenotypes identical to the *hd1* mutant, with 7.7 days (LD) or 2.0 days (SD) earlier than the *jmj720* mutants (Figure 7c–e), demonstrating complete epistasis of Hd1 over both JMJ720 and POH1. These genetic interactions establish that JMJ720, POH1 and Hd1 might function in the same genetic pathway regulating flowering, with Hd1 acting downstream of JMJ720 and POH1.

### Global distribution patterns of JMJ720 haplotypes

**Figure 8:**
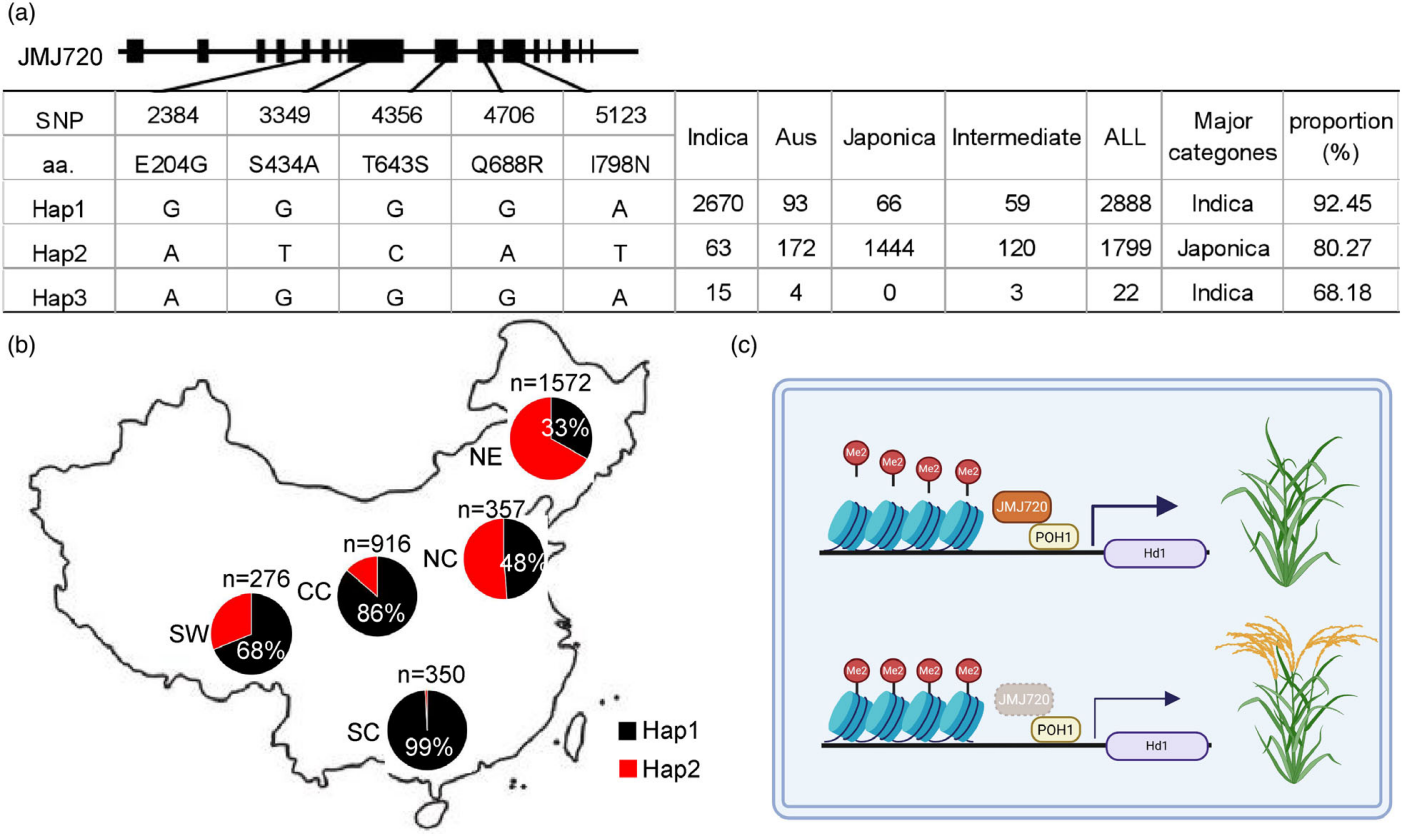
Natural variation and working model for JMJ720. (a) Diagram showing the SNP representing three haplotypes based on the five polymorphic sites. The black bars denote exons. Polymorphic nucleotides and the amino acid (aa) substitutions are indicated. The number of the JMJ720 for 4709 diverse Asian cultivated rice is from the Rice Variation Map database. (b) Geographical distribution of 4426 rice landraces carrying different JMJ720 haplotypes from RiceAtlas database. CC, Central China; NC, North China; NE, Northeast; SC, South China; SW, Southwest Plateau. (c) Working model for the flowering control of histone demethylations by JMJ720. POH1 transcription factor acts as a transcription activator and recruits JMJ720 to remove H3K9me2 from the *Hd1* locus, resulting in increased *Hd1* expression.

Given the haplotypes variation of JMJ720 in TH899 and K4, we conducted extensive polymorphism screening to characterize JMJ720 genetic diversity across diverse germplasm. Analysis of the Rice SNP‐Seek Database (https://snp‐seek.irri.org/) revealed that JMJ720 exists primarily in three haplotypes (Hap1, Hap2 and Hap3) among rice varieties, distinguished by five amino acid substitutions (Figure 8a). Then we utilized the Rice Variation Map to analyse haplotype networks in the world. We found Hap1 was predominantly found in *indica*, accounting for 92.45% of the total, while Hap2 is mainly present in *japonica*, making up 80.27% of the total. Hap3 was absent in *japonica* but occurred at low frequencies in *indica*, *Aus* and *intermediate* varieties (Figure 8a). Further examination of 4426 collected varieties from the RiceAtlas database showed a widespread distribution of Hap1 and Hap2 across China. South China exclusively harbors Hap1 (99%). Northeast China is dominated by Hap2 (67%) and Central China exhibits a high prevalence of Hap1 (86%) (Figure 8b). Notably, no significant difference in heading date was observed between Hap1 and Hap2 across diverse regions, supporting that JMJ720 is a minor flowering regulator (Figure S10).

## Discussion

### JMJ720 is an H3K9me2 demethylase regulating flowering time in rice

Histone methylation serves as a critical epigenetic mechanism governing plant growth and development through its dual regulation of chromatin architecture and gene expression (Chen *et al*., 2011). The precise equilibrium between histone methylation and demethylation, mediated by specific methyltransferases and demethylases, constitutes a fundamental determinant of flowering time. JMJ720 has been classified as a member of a jumonji domain‐containing histone demethylases of the JHDM2 group (Lu *et al*., 2008; Sun and Zhou, 2008). In this study, we found JMJ720 is a novel epigenetic regulator of flowering time in rice. Loss‐of‐function *jmj720* mutants exhibited an early flowering phenotype (Figure 2). JMJ720 demethylates H3K9me2 based on *in vitro* and *in vivo* assays, and the level of H3K9me2 was increased in *jmj720* mutants and was decreased in *JMJ720 OE* (Figure 4a,b). The differential enzymatic activity between TH899 and K4 variants results from five amino acid substitutions (Figures 1c and 4c).

In *Arabidopsis*, most methyltransferases and demethylases are involved in flowering regulation by modulating H3 lysine 4 (H3K4) or H3 lysine 27 (H3K27) methylation levels. For instance, SDG25 and SDG27 target *FLOWERING LOCUS C* (*FLC*) by H3K4 methylation to repress flowering, while JMJ15 and JMJ18 function as H3K4 demethylases at the *FLC* locus to accelerate flowering time (Yang *et al*., 2012a,b). Despite both being H3K36 methyltransferases, SDG8 inhibits flowering via activating *FLC*, while SDG26 accelerates flowering by activating *SOC1*. JMJ30, JMJ32, EARLY FLOWERING 6 (ELF6/JMJ11) and RELATIVE OF EARLY FLOWERING 6 (REF6/JMJ12) all have H3K27me3 demethylation activity by targeting the *FLC* locus, and then play divergent roles in the regulation of flowering (Crevillen *et al*., 2014; Noh *et al*., 2004). JMJ27 and JMJ28 play different roles in the regulation of flowering by H3K9 demethylation (Dutta *et al*., 2017; Hung *et al*., 2021).

In rice, several SDG methyltransferases have been shown to regulate flowering time. As H3K4‐specific methyltransferases, SDG701 and OxTrx1/SDG723 promote flowering by altering H3K4me3 levels at florigens or *Ehd1* (Choi *et al*., 2014; Jiang *et al*., 2018; Liu *et al*., 2017). SDG708, SDG724 and SDG725 act as H3K36‐specific methyltransferases, promoting flowering by inducing the expression of *Ehd1*, *Hd3a* and *RFT1* (Liu *et al*., 2016; Sui *et al*., 2013; Sun *et al*., 2012). Both SDG711 and SDG718 act as H3K27‐specific methyltransferases, playing divergent roles in flowering in LD and SD (Liu *et al*., 2014b). Notably, only SDG712 functions downstream of *Hd1* and upstream of *Ehd1*, *Hd3a* and *RFT1* by mediating H3K9me2 at these loci to delay flowering (Zhang *et al*., 2021). In addition to the methylation enzymes described above, just two demethylases have been identified as flowering regulators in rice. Se14 suppresses flowering through the demethylation of H3K4me3 of *RFT1* under LD (Yokoo *et al*., 2014). JMJ706/ISPL10 interacted with Se14 to repress *RFT1* expression by demethylating H3K4me3 at the *RFT1* locus. Additionally, ISPL10 activated *OsMADS51* expression by removing H3K9me2 at the *OsMADS51* locus (Yu *et al*., 2025). JMJ720 has histone H3K9 demethylase activity that differs from SDG712, as JMJ720 cannot concurrently regulate H3K9me2 levels at the *Ehd1*, *Hd3a* and *RFT1* loci (Figure 5). In summary, our study identifies JMJ720 as a novel epigenetic regulator of flowering in rice that modulates *Hd1* expression through H3K9me2 modification.

### POH1 delays heading date in a JMJ720‐dependent manner

Histone modifications play important roles in various biological processes including inflorescence development and stress responses. While some modifications function independently, others operate through coordinated regulatory complexes. JMJ706 is recruited by WOX11 to enhance *LBD16* transcription, thereby regulating rice crown root development (Geng *et al*., 2024). Recent findings further demonstrate that JMJ706/ISPL10 interacts with the SDG701/Se14 to modulate H3K4me2 methylation levels at the *RFT1* locus (Yu *et al*., 2025). In rice shoot growth, WOX11 recruits JMJ705 to remove H3K27me3 marks from genes involved in developmental and energy metabolism pathways (Cheng *et al*., 2018). BES1 recruits ELF6 and REF6 to activate BR signalling and promote cell elongation (Yu *et al*., 2008). SDG711 and JMJ703 function synergistically in regulating histone methylation and gene expression within the inflorescence meristem (Liu *et al*., 2015). These demethylases may be recruited by different transcription factors or target distinct flowering‐related genes, potentially explaining their opposing effects on flowering time regulation.

POH1 has been experimentally confirmed to bind the promoter region of *Hd1* and activate its expression (Yin *et al*., 2023). In this study, our ChIP experiments further verified POH1 could bind to the *Hd1* promoter (Figure S8). Meanwhile, the H3K9me2 level was increased in the promoter region at the *Hd1* locus in *jmj720‐3* and *jmj720‐3 POH1 OE*, but maintained unchanged in *POH1 OE‐1* (Figures 5b and 7b). Collectively, these results demonstrate that POH1 might serve not only as a transcriptional activator but can also recruit JMJ720 to reduce H3K9me2 levels at the *Hd1* locus. Therefore, based on our findings, we propose the model illustrated in Figure 8c: in WT plants, POH1 reduces H3K9me2 methylation at the *Hd1* locus by recruiting the histone demethylase JMJ720, thereby enhancing *Hd1* expression and consequently repressing flowering. In contrast, *jmj720* mutants exhibit elevated H3K9me2 levels at the *Hd1* locus, which interferes with POH1‐mediated transcriptional activation. This leads to reduced *Hd1* expression and results in an early flowering phenotype. In *Arabidopsis*, four FBHs recruit JMJ28 to promote flowering by activating *CO* expression (Ito *et al*., 2012). We showed that POH1 recruits JMJ720 to regulate the H3K9me2 modification of *Hd1*, and delay flowering (Figure 8c). Interestingly, *FBH* and *POH1*, *JMJ28* and *JMJ720*, and CO and *Hd1*, are corresponding orthologous between *Arabidopsis* and rice. These results imply the existence of this conserved flowering time regulation mechanism across diverse species.

Hd1, as an important factor in regulating flowering time of rice, has dual functions in controlling flowering time in rice. In this study, we generated *hd1* mutants in the ZH11 background. Phenotypic characterization revealed early flowering in both LD and SD conditions, with more pronounced early flowering under LD (Figure 7e). This observation aligns with previous reports that *Hd1* overexpression inhibits flowering under both SD and LD conditions (Ishikawa *et al*., 2011), knockout of *Hd1* produced extremely early heading phenotype (Zhou *et al*., 2024). Furthermore, the late‐flowering phenotype of *haf1* mutants (where HAF1 targets Hd1 for degradation) indirectly supports that *Hd1* is a flowering repressor (Yang *et al*., 2015). While a high degree of polymorphism in the *Hd1* coding sequence and the formation of the Hd1‐Ghd7, Hd1–DTH8 protein complex, it was difficult to accurately distinguish functional proteins from nonfunctional proteins (Du *et al*., 2017; Nemoto *et al*., 2016; Takahashi *et al*., 2009). At the post‐translational level, Hd1 activity is phosphorylated by OsK4 kinase, which potentially regulates Hd1 protein stability (Sun *et al*., 2016). OsSFL1‐mediated histone deacetylation at the *Hd1* locus leads to suppressed *Hd3a* expression under SD conditions (Geng *et al*., 2020). This study reveals JMJ720 as a novel epigenetic regulator that catalyses H3K9me2 demethylation at the *Hd1* locus. In *jmj720* mutants, elevated H3K9me2 modification levels at *Hd1* (Figure 5b) correlate with reduced expression and consequent early flowering phenotype (Figure 2). Phenotypic analysis revealed that the *hd1* mutants exhibited earlier flowering under both LD and SD conditions (Figure 7c,e). This observation aligns with evidence that Hd1 functions as a flowering repressor under both SD and LD conditions (Ishikawa *et al*., 2011; Zhou *et al*., 2024). Furthermore, the flowering regulatory function of Hd1 depends on the *Ghd7* genotype, as Ghd7 is functional in TH899 and ZH11, so Hd1 inhibits flowering under both LD and SD conditions (Nemoto *et al*., 2016).

### Importance of minor‐effect QTLs in crop adaptation and breeding

Maximum regional adaptability and yield potential are closely linked to crop heading date, a critical determinant of agricultural success (Guo *et al*., 2020). In rice, yield exhibits a strong positive correlation with heading date (Sun *et al*., 2022). Natural variations exist in key flowering regulators such as *Hd1*, *Ghd7*, *DTH8* and *PRR37*, with different allele combinations resulting in diverse heading dates and photoperiod sensitivity (Sun *et al*., 2022; Zhang *et al*., 2019a; Zong *et al*., 2021). However, many cultivars exhibit distinct flowering despite sharing similar core flowering regulators, suggesting that there are other shatter‐controlling genes differentially fixed within different subpopulations of rice (Ma *et al*., 2025). Our comprehensive analysis of JMJ720 haplotypes variation revealed three major haplotypes (Hap1, Hap2 and Hap3) distinguished by five amino acid substitutions (Figure 8a). Strikingly, these haplotypes exhibit divergence between *indica* and *japonica*, suggesting potential functional constraints or differential selection pressures acting on JMJ720 across rice subpopulations (Figure 8a). Notably, despite these strong haplotype‐divergence patterns, no significant differences in heading date were observed between Hap1 and Hap2 across regions (Figure S10). This suggests that JMJ720 may play only a minor role in flowering time regulation, consistent with its classification as a fine‐tuning epigenetic modifier rather than a major flowering pathway component. Additionally, the distribution patterns of these accessions may also be associated with the enzymatic activity differences between the two JMJ720 haplotypes (Figure 4c). GST‐JMJ720‐TH899 displayed enhanced demethylase activity relative to GST‐JMJ720‐K4, suggesting those five amino acid substitutions between TH899 and K4 indeed affect its demethylation activity (Figure 4c). Thus, we analyse these five variant sites on the rice variation map (ricevarmap.ncpgr.cn) website. Based on the data of the five SNPs displayed on the website, combined with functional prediction tools and chromatin accessibility scores, the SNPs were prioritized in the following order (from highest to lowest) I798N > T643S > S434A > Q688R > E204G. Therefore, I798N and T643S are the most likely key variants causing the functional change of JMJ720. We will test the function of I798N and T643S via single‐base editing in the future. However, the strong haplotype‐subspecies associations raise intriguing questions about whether JMJ720 variation contributes to other adaptive traits, such as stress responses or yield‐related processes, independent of flowering time. Future functional studies comparing near‐isogenic lines carrying different JMJ720 haplotypes could clarify its pleiotropic effects beyond flowering regulation.

In conclusion, this study identifies JMJ720 as a novel flowering time regulator in rice that functions through a POH1‐JMJ720‐Hd1 epigenetic regulatory module. We demonstrate that JMJ720, as an H3K9me2‐specific demethylase, is recruited by POH1 to remove repressive marks at the *Hd1* locus, thereby enabling *Hd1*‐mediated flowering repression. The natural variation in JMJ720 coding sequence between TH899 and K4 leads to differential demethylase activity, providing a molecular basis for flowering time differences. Despite showing strong subspecies‐specific haplotype distributions, JMJ720 haplotypes do not significantly affect heading date, consistent with its role as a minor flowering regulator. Importantly, *jmj720* mutants exhibit early flowering without yield penalty, offering valuable genetic material for breeding programs. Our findings not only expand our understanding of histone demethylase functions in flowering control but also provide novel insights into epigenetic fine‐tuning of agronomically important traits.

## Materials and methods

### Plant materials and growth conditions

Rice cultivar Tonghe899 (TH899), K4 and Zhonghua 11 (ZH11) were used as wild type controls to compare with diverse mutants, complemented or overexpression transgenic plants in relative background. Plants were grown in a greenhouse in Harbin (45° N) under long‐day (LD) conditions (14‐h light/10‐h dark at 28 °C) or short‐day (SD) conditions (10‐h light/14‐h dark). The heading date was recorded when the first panicle became visible.

### Construction of plasmids

To generate a 1300‐JMJ720gDNA construct, the *JMJ720* promoter region and *JMJ720* genomic DNA sequences were amplified using 1300‐gDNA‐JMJ720‐F/R primers. Subsequently, they were inserted into the binary vector *pCAMBIA1300*. To generate a *JMJ720*_*Pro*_ *‐GUS* construct, the 1951 bp of the *JMJ720* promoter region was inserted into the pENTRY vector and subcloned into the *pHGWFS7* vector through the LR reaction. *JMJ720*, *POH1* or *Hd1* cDNA was isolated from ZH11 by PCR using the gene‐specific primers. To generate a *35S*_*Pro*_ *:GFP‐JMJ720* construct, *JMJ720* was introduced into the pENTRY vector and cloned into *pH7WGF2* through the LR reaction. To generate GST‐JMJ720 and MBP‐POH1 constructs, *JMJ720* and *POH1* were cloned into the *pGEX4T* vector and *pMAL‐c2X* vector respectively. All primer sequences are listed in Table S1.

### Quantitative real‐time RT‐PCR

To analyse diurnal expression patterns by RT‐qPCR, TH899 and *jmj720‐1* plants were grown for 40 days under LD or SD conditions, with samples collected every 4 h starting from the onset of the light period. For overexpression analysis, wild‐type (WT) and transgenic plants at identical developmental stages were harvested. Total RNA was extracted using Trizol reagent (Invitrogen), and 1.5 μg of RNA was reverse transcribed into cDNA using Superscript II Reverse Transcriptase (Invitrogen). Real‐time PCR was performed with SYBR Green PCR master mix (TransGen) on a LightCycler480 II system. The *Ubiquitin* gene (Os01g0328400) served as an internal reference, and relative expression levels were calculated using the 2^−ΔCt^ method. Primer sequences are provided in Table S1.

### GUS staining

Various tissues from different developmental stages of *JMJ720*_*Pro*_ *‐GUS* transgenic plants were collected and immersed in GUS staining solution. The staining buffer consisted of 50 mM sodium phosphate buffer, pH 7.0, 0.5 mM potassium ferrocyanide, 0.5 mM potassium ferricyanide and 0.5 mg/ml X‐glucuronide. After incubation at room temperature overnight in the dark, samples were destained sequentially with 95% and 75% ethanol before imaging.

### Subcellular localization

The JMJ720 coding sequence was fused with GFP to generate the recombinant vector *35S*_*Pro*_ *:GFP‐JMJ720*, which was subsequently transformed into *Agrobacterium* strain GV3101. Bacterial cultures were grown overnight in medium containing 10 mM MES (pH 5.6) and 20 μM acetosyringone, harvested, and resuspended in infiltration buffer (10 mM MgCl_2_ with 100 μM acetosyringone) to an OD_600_ of 1.5. The suspension was co‐infiltrated into young *N. benthamiana* leaves. After 40–48 h infiltration, leaf samples were stained with 4′,6‐diamidino‐2‐phenylindole (DAPI) for 5 min at room temperature. GFP and DAPI fluorescence were observed by confocal microscopy (Leica).

### Histone demethylation assay *in vivo*

Histone‐enriched protein fractions were extracted from rice leaves by Plant Histone Extraction Kit (BestBio; BB‐31171‐50T). Histone methylation modification was detected by Western blotting with anti‐H3K9me2 antibody (ABclonal, A2359). The H3 protein was detected by anti‐H3 antibody (Abcam, ab1791). Other antibodies were H3K9me1 (Millipore, 07‐450), H3K4me1 (Millipore, 07‐436), H3K4me2 (Millipore, 07‐790), H3K4me3 (Millipore, 07‐473), H3K27me1 (Millipore, 07‐448), H3K27me2 (Millipore, 07‐452), H3K27me3 (Abmart, ab6002), H3K36me1 (Abcam, ab9048), H3K36me2 (Millipore, 07‐274) and H3K36me3 (Abcam, ab9050). Relative protein levels in different lanes were quantified using ImageJ software.

### Histone demethylation assay *in vitro*

The coding sequences of JMJ720 from TH899 and K4 were individually fused with GST and transformed into *E. coli* BL21. Recombinant fusion proteins were affinity‐purified using glutathione‐agarose beads. Equal amounts of purified proteins were incubated with calf thymus histones (H9250; Sigma‐Aldrich) in reaction buffer (20 mM Tris‐Cl, pH 7.5, 150 mM NaCl, 50 μM Fe(NH_4_)_2_(SO_4_)_2_, 1 mM α‐ketoglutaric acid, 2 mM ascorbic acid) for 4 h at 37 °C. Reaction products were then analysed by Western blot using anti‐H3K9me2 and anti‐H3 antibodies.

### Yeast two‐hybrid assays

For the yeast two‐hybrid, the coding sequences of JMJ720 were cloned into the pGBKT7 vector, while the coding sequences of POH1 and Hd1 were cloned into the pGADT7 vector. These recombinant vectors, along with empty pGADT7 and pGBKT7 controls, were co‐transformed into the yeast strain Y2H Gold. Transformants were incubated on the SD/‐Trp/‐Leu medium at 28 °C for 3 days. Colonies were then transferred onto SD/‐Ade/‐His/‐Leu/‐Trp medium to test for protein–protein interactions.

### *In vitro* pull‐down assays

The coding sequences of POH1 were fused with MBP (*pMAL‐c2X*) along with empty MBP and GST‐JMJ720 transformed into *E. coli* BL21. GST‐JMJ720 was incubated with MBP‐POH1 or MBP on GST beads and pulled down from the GST beads. The proteins were subsequently analysed by immunoblot with anti‐GST antibody (Mouse, ABclonal, M20007M, 1:2000 dilution) and anti‐MBP antibody (Mouse, Abmart, AE016, 1:2000 dilution).

### Split‐LUC complementation assays

The coding regions of JMJ720 were cloned into the cLUC fragment, while POH1 and Hd1 were cloned into the nLUC fragment of the *pCAMBIA1300* vector. Different combinations of these constructs were transiently co‐expressed in *N. benthamiana* leaves via *Agroinfiltration*. LUC activity was assessed 48 h post‐infiltration using a Tanon 5200 chemiluminescence imaging system with 1 mM D‐luciferin potassium salt as substrate.

### Co‐immunoprecipitation analysis

The JMJ720 coding sequence was cloned into *pCambia1300‐221‐3xFlag* vector and the POH1 coding sequence was cloned into *pCambia1300‐221‐6xMyc* vector. To resolve the comigration of POH1 with immunoglobulin heavy chains during immunoblotting, we incorporated GFP into the recombinant vector to increase the apparent molecular weight, using GFP‐Flag as a control. Rice protoplasts were cotransformed with either JMJ720‐Flag or GFP‐Flag constructs together with POH1‐GFP‐Myc. After 16 h of cocultivation, total proteins were extracted using lysis buffer (50 mM Tris–HCl, pH 7.5, 150 mM NaCl, 0.5 mM EDTA, pH 8.0, 10% glycerol, 0.5% Triton X‐100). JMJ720‐Flag and GFP‐Flag were immunoprecipitated using anti‐Flag agarose beads. Immunoprecipitated proteins were analysed by immunoblot with anti‐Flag (Mouse, Abmart, M20118, 1:2000 dilution) and anti‐Myc (Mouse, ThermoFisher, 9E10, 1:2000 dilution; Sigma) antibodies.

### Chromatin immunoprecipitation assays

Chromatin immunoprecipitation was performed according to the method as previously described (Tian *et al*., 2017). Briefly, approximately 2 g of leaf tissue were harvested and vacuum‐infiltrated with 1% formaldehyde for 30 min to cross‐link proteins to DNA. Cross‐linked tissues were ground to a fine powder in liquid nitrogen, followed by nuclei isolation. All samples were processed by extraction and sonication, followed by collection of supernatants. Anti‐Myc and anti‐H3K9me2 were used to immunoprecipitate the protein–DNA complex. The antibody‐protein complexes were isolated by binding to protein A beads. Chromatin precipitated without antibody served as a negative control. ChIP‐qPCR was conducted to analyse DNA modifications in the immunoprecipitated chromatin.

## Author contributions

Q.B. conceived and designed the experiments. X.L. performed most of the experiments with the help from J.Z., X.T., Z.H., X.J., C.C., J.W., Y.L., Z.W. and J.T. Q.B. and X.L. analysed the data and wrote the paper. X.S. revised the manuscript. The authors read and approved the final manuscript.

## Conflict of interest

The authors declare that they have no conflict of interest.

## Consent for publication

Not applicable.

## Supporting information


**Figure S1** Identification of *JMJ720*.
**Figure S2** The *jmj720* mutant in the ZH11 background accelerates rice flowering.
**Figure S3** The *jmj720* mutants exhibited no significant differences in agronomic traits compared to TH899.
**Figure S4** JMJ720 regulates flowering‐related gene expression.
**Figure S5** *JMJ720 OE* does not change the flowering in the ZH11 background.
**Figure S6** JMJ720 does not interact with Hd1.
**Figure S7** Characterization of *POH OE* plants in ZH11 background and *jmj720‐3* mutant.
**Figure S8** ChIP assays showing that the enrichment of *Hd1* in *POH1 OE* plants.
**Figure S9** Characterization of *poh1*, *hd1 jmj720* and *hd1 jmj720 poh1* mutants in ZH11 background.
**Figure S10** Association analysis of JMJ720 haplotypes with flowering time in rice germplasm from RiceAtlas database.

**Table S1** Primers used in this paper.

## Data Availability

The data that support the findings of this study are available in the Supporting Information of this article.

## References

[pbi70206-bib-0001] Chen, X. , Hu, Y. and Zhou, D.X. (2011) Epigenetic gene regulation by plant Jumonji group of histone demethylase. Biochim. Biophys. Acta, 1809, 421–426. 21419882 10.1016/j.bbagrm.2011.03.004

[pbi70206-bib-0002] Cheng, S. , Tan, F. , Lu, Y. , Liu, X. , Li, T. , Yuan, W. , Zhao, Y. *et al*. (2018) WOX11 recruits a histone H3K27me3 demethylase to promote gene expression during shoot development in rice. Nucleic Acids Res. 46, 2356–2369. 29361035 10.1093/nar/gky017 PMC5861455

[pbi70206-bib-0003] Choi, S.C. , Lee, S. , Kim, S.R. , Lee, Y.S. , Liu, C. , Cao, X. and An, G. (2014) Trithorax group protein *Oryza sativa* Trithorax1 controls flowering time in rice via interaction with early heading date3. Plant Physiol. 164, 1326–1337. 24420930 10.1104/pp.113.228049 PMC3938623

[pbi70206-bib-0004] Crevillen, P. , Yang, H. , Cui, X. , Greeff, C. , Trick, M. , Qiu, Q. , Cao, X. *et al*. (2014) Epigenetic reprogramming that prevents transgenerational inheritance of the vernalized state. Nature, 515, 587–590. 25219852 10.1038/nature13722 PMC4247276

[pbi70206-bib-0005] Du, A. , Tian, W. , Wei, M. , Yan, W. , He, H. , Zhou, D. , Huang, X. *et al*. (2017) The DTH8‐Hd1 module mediates day‐length‐dependent regulation of rice flowering. Mol. Plant, 10, 948–961. 28549969 10.1016/j.molp.2017.05.006

[pbi70206-bib-0006] Dutta, A. , Choudhary, P. , Caruana, J. and Raina, R. (2017) JMJ27, an Arabidopsis H3K9 histone demethylase, modulates defense against *Pseudomonas syringae* and flowering time. Plant J. 91, 1015–1028. 28650521 10.1111/tpj.13623

[pbi70206-bib-0008] Geng, L. , Tan, M. , Deng, Q. , Wang, Y. , Zhang, T. , Hu, X. , Ye, M. *et al*. (2024) Transcription factors WOX11 and LBD16 function with histone demethylase JMJ706 to control crown root development in rice. Plant Cell, 36, 1777–1790. 38190205 10.1093/plcell/koad318 PMC11062443

[pbi70206-bib-0007] Geng, Y. , Zhang, P. , Liu, Q. , Wei, Z. , Riaz, A. , Chachar, S. and Gu, X. (2020) Rice homolog of Sin3‐associated polypeptide 30, OsSFL1, mediates histone deacetylation to regulate flowering time during short days. Plant Biotechnol. J. 18, 325–327. 31446676 10.1111/pbi.13235 PMC6953189

[pbi70206-bib-0009] Guo, T. , Mu, Q. , Wang, J. , Vanous, A.E. , Onogi, A. , Iwata, H. , Li, X. *et al*. (2020) Dynamic effects of interacting genes underlying rice flowering‐time phenotypic plasticity and global adaptation. Genome Res. 30, 673–683. 32299830 10.1101/gr.255703.119 PMC7263186

[pbi70206-bib-0010] Hori, K. , Matsubara, K. and Yano, M. (2016) Genetic control of flowering time in rice: integration of Mendelian genetics and genomics. Theor. Appl. Genet. 129, 2241–2252. 27695876 10.1007/s00122-016-2773-4

[pbi70206-bib-0011] Hu, Z. , Yang, Z.P. , Zhang, Y. , Zhang, A.H. , Lu, Q.T. , Fang, Y. and Lu, C.M. (2022) Autophagy targets Hd1 for vacuolar degradation to regulate rice flowering. Mol. Plant, 15, 1137–1156. 35591785 10.1016/j.molp.2022.05.006

[pbi70206-bib-0012] Huang, S.Z. , Zhang, A. , Jin, J.B. , Zhao, B. , Wang, T.J. , Wu, Y.F. , Wang, S. *et al*. (2019) Histone H3K4 demethylase JMJ17 functions in dehydration stress response. New Phytol. 223, 1372–1387. 31038749 10.1111/nph.15874

[pbi70206-bib-0013] Hung, F.Y. , Lai, Y.C. , Wang, J. , Feng, Y.R. , Shih, Y.H. , Chen, J.H. , Sun, H.C. *et al*. (2021) The Arabidopsis histone demethylase JMJ28 regulates CONSTANS by interacting with FBH transcription factors. Plant Cell, 33, 1196–1211. 33604650 10.1093/plcell/koab014

[pbi70206-bib-0014] Ishikawa, R. , Aoki, M. , Kurotani, K. , Yokoi, S. , Shinomura, T. , Takano, M. and Shimamoto, K. (2011) Phytochrome B regulates Heading date 1 (Hd1)‐mediated expression of rice florigen Hd3a and critical day length in rice. Mol. Gen. Genomics., 285, 461–470. 10.1007/s00438-011-0621-4 21512732

[pbi70206-bib-0015] Ito, S. , Song, Y.H. , Josephson‐Day, A.R. , Miller, R.J. , Breton, G. , Olmstead, R.G. and Imaizumi, T. (2012) FLOWERING BHLH transcriptional activators control expression of the photoperiodic flowering regulator CONSTANS in Arabidopsis. Proc. Natl. Acad. Sci. USA, 109, 3582–3587. 22334645 10.1073/pnas.1118876109 PMC3295255

[pbi70206-bib-0016] Jeong, J.H. , Song, H.R. , Ko, J.H. , Jeong, Y.M. , Kwon, Y.E. , Seol, J.H. , Amasino, R.M. *et al*. (2009) Repression of FLOWERING LOCUS T chromatin by functionally redundant histone H3 lysine 4 demethylases in Arabidopsis. PLoS One, 4, e8033. 19946624 10.1371/journal.pone.0008033 PMC2777508

[pbi70206-bib-0017] Jiang, P.F. , Wang, S.L. , Zheng, H. , Li, H. , Zhang, F. , Su, Y.H. , Xu, Z.T. *et al*. (2018) SIP1 participates in regulation of flowering time in rice by recruiting OsTrx1 to Ehd1. New Phytol. 219, 422–435. 29611871 10.1111/nph.15122 PMC6001661

[pbi70206-bib-0018] Klose, R.J. , Kallin, E.M. and Zhang, Y. (2006) JmjC‐domain‐containing proteins and histone demethylation. Nat. Rev. Genet. 7, 715–727. 16983801 10.1038/nrg1945

[pbi70206-bib-0019] Kooistra, S.M. and Helin, K. (2012) Molecular mechanisms and potential functions of histone demethylases. Nat. Rev. Mol. Cell Biol. 13, 297–311. 22473470 10.1038/nrm3327

[pbi70206-bib-0020] Li, X. , Zhu, B. , Lu, Y. , Zhao, F. , Liu, Q. , Wang, J.H. , Ye, M.M. *et al*. (2024) DNA methylation remodeling and the functional implication during male gametogenesis in rice. Genome Biol. 25, 84. 38566207 10.1186/s13059-024-03222-w PMC10985897

[pbi70206-bib-0024] Liu, B. , Wei, G. , Shi, J.L. , Jin, J. , Shen, T. , Ni, T. , Shen, W.H. *et al*. (2016) SET DOMAIN GROUP 708, a histone H3 lysine 36‐specific methyltransferase, controls flowering time in rice (*Oryza sativa*). New Phytol. 210, 577–588. 26639303 10.1111/nph.13768

[pbi70206-bib-0021] Liu, H. , Guo, S. , Xu, Y. , Li, C. , Zhang, Z. , Zhang, D. , Xu, S. *et al*. (2014a) OsmiR396d‐regulated OsGRFs function in floral organogenesis in rice through binding to their targets OsJMJ706 and OsCR4. Plant Physiol. 165, 160–174. 24596329 10.1104/pp.114.235564 PMC4012577

[pbi70206-bib-0025] Liu, K.P. , Yu, Y. , Dong, A.W. and Shen, W.H. (2017) SET DOMAIN GROUP701 encodes a H3K4‐methytransferase and regulates multiple key processes of rice plant development. New Phytol. 215, 609–623. 28517045 10.1111/nph.14596

[pbi70206-bib-0023] Liu, X. , Zhou, S. , Wang, W. , Ye, Y. , Zhao, Y. , Xu, Q. , Zhou, C. *et al*. (2015) Regulation of histone methylation and reprogramming of gene expression in the rice inflorescence meristem. Plant Cell, 27, 1428–1444. 25957386 10.1105/tpc.15.00201 PMC4456649

[pbi70206-bib-0022] Liu, X.Y. , Zhou, C. , Zhao, Y. , Zhou, S.L. , Wang, W.T. and Zhou, D.X. (2014b) The rice enhancer of zeste [E(z)] genes SDG711 and SDG718 are respectively involved in long day and short day signaling to mediate the accurate photoperiod control of flowering time. Front. Plant Sci. 5, 1–9. 10.3389/fpls.2014.00591 PMC4215622 25400654

[pbi70206-bib-0027] Lu, F. , Cui, X. , Zhang, S. , Liu, C. and Cao, X. (2010) JMJ14 is an H3K4 demethylase regulating flowering time in Arabidopsis. Cell Res. 20, 387–390. 20177424 10.1038/cr.2010.27

[pbi70206-bib-0026] Lu, F. , Li, G. , Cui, X. , Liu, C. , Wang, X.J. and Cao, X. (2008) Comparative analysis of JmjC domain‐containing proteins reveals the potential histone demethylases in Arabidopsis and rice. J. Integr. Plant Biol. 50, 886–896. 18713399 10.1111/j.1744-7909.2008.00692.x

[pbi70206-bib-0028] Ma, X. , Wang, H. , Yan, S. , Zhou, C. , Zhou, K. , Zhang, Q. , Li, M. *et al*. (2025) Large‐scale genomic and phenomic analyses of modern cultivars empower future rice breeding design. Mol. Plant, 18, 651–668. 40083159 10.1016/j.molp.2025.03.007

[pbi70206-bib-0029] Nemoto, Y. , Nonoue, Y. , Yano, M. and Izawa, T. (2016) Hd1,a CONSTANS ortholog in rice, functions as an Ehd1 repressor through interaction with monocot‐specific CCT‐domain protein Ghd7. Plant J. 86, 221–233. 26991872 10.1111/tpj.13168

[pbi70206-bib-0030] Noh, B. , Lee, S.H. , Kim, H.J. , Yi, G. , Shin, E.A. , Lee, M. , Jung, K.J. *et al*. (2004) Divergent roles of a pair of homologous jumonji/zinc‐finger‐class transcription factor proteins in the regulation of Arabidopsis flowering time. Plant Cell, 16, 2601–2613. 15377760 10.1105/tpc.104.025353 PMC520958

[pbi70206-bib-0031] Qiu, L.L. , Wu, Q.Q. , Wang, X.Y. , Han, J.P. , Zhuang, G. , Wang, H. , Shang, Z.Y. *et al*. (2021) Forecasting rice latitude adaptation through a daylength‐sensing‐based environment adaptation simulator. Nat. Food, 2, 348. 37117734 10.1038/s43016-021-00280-2

[pbi70206-bib-0032] Sui, P.F. , Shi, J.L. , Gao, X.Y. , Shen, W.H. and Dong, A.W. (2013) H3K36 methylation is involved in promoting rice flowering. Mol. Plant, 6, 975–977. 23239829 10.1093/mp/sss152

[pbi70206-bib-0034] Sun, C. , Fang, J. , Zhao, T. , Xu, B. , Zhang, F. , Liu, L. , Tang, J. *et al*. (2012) The histone methyltransferase SDG724 mediates H3K36me2/3 deposition at MADS50 and RFT1 and promotes flowering in rice. Plant Cell, 24, 3235–3247. 22892321 10.1105/tpc.112.101436 PMC3462628

[pbi70206-bib-0037] Sun, K. , Zong, W. , Xiao, D. , Wu, Z. , Guo, X. , Li, F. , Song, Y. *et al*. (2023) Effects of the core heading date genes Hd1, Ghd7, DTH8, and PRR37 on yield‐related traits in rice. Theor. Appl. Genet. 136, 227. 37851149 10.1007/s00122-023-04476-x

[pbi70206-bib-0036] Sun, K.L. , Huang, M.H. , Zong, W.B. , Xiao, D.D. , Lei, C. , Luo, Y.Q. , Song, Y.G. *et al*. (2022) Hd1, Ghd7, and DTH8 synergistically determine the rice heading date and yield‐related agronomic traits. J. Genet. Genomics, 49, 437–447. 35248762 10.1016/j.jgg.2022.02.018

[pbi70206-bib-0033] Sun, Q. and Zhou, D.X. (2008) Rice jmjC domain‐containing gene JMJ706 encodes H3K9 demethylase required for floral organ development. Proc. Natl. Acad. Sci. USA, 105, 13679–13684. 18765808 10.1073/pnas.0805901105 PMC2533249

[pbi70206-bib-0038] Sun, R. , Ding, Y. , Mimura, M. , Nishide, N. and Izawa, T. (2025) Temporal transcriptome analysis reveals the two‐phase action of florigens in rice flowering. Theor. Appl. Genet. 138, 100. 40220150 10.1007/s00122-025-04869-0 PMC11993458

[pbi70206-bib-0035] Sun, X. , Zhang, Z. , Wu, J. , Cui, X. , Feng, D. , Wang, K. , Xu, M. *et al*. (2016) The *Oryza sativa* regulator HDR1 associates with the kinase OsK4 to control photoperiodic flowering. PLoS Genet. 12, e1005927. 26954091 10.1371/journal.pgen.1005927 PMC4783006

[pbi70206-bib-0039] Takahashi, Y. , Teshima, K.M. , Yokoi, S. , Innan, H. and Shimamoto, K. (2009) Variations in Hd1 proteins, Hd3a promoters, and Ehd1 expression levels contribute to diversity of flowering time in cultivated rice. Proc. Natl. Acad. Sci. USA, 106, 4555–4560. 19246394 10.1073/pnas.0812092106 PMC2647979

[pbi70206-bib-0055] Tian, X. , Li, X. , Zhou, W. , Ren, Y. , Wang, Z. , Liu, Z. , *et al*. (2017) Transcription factor OsWRKY53 positively regulates brassinosteroid signaling and plant architecture. Plant physiology, 175(3), 1337–1349. 28894020 10.1104/pp.17.00946 PMC5664471

[pbi70206-bib-0040] Wei, X.J. , Xu, J.F. , Guo, H.N. , Jiang, L. , Chen, S.H. , Yu, C.Y. , Zhou, Z.L. *et al*. (2010) Suppresses flowering in rice, influencing plant height and yield potential simultaneously. Plant Physiol. 153, 1747–1758. 20566706 10.1104/pp.110.156943 PMC2923886

[pbi70206-bib-0041] Yan, W.H. , Wang, P. , Chen, H.X. , Zhou, H.J. , Li, Q.P. , Wang, C.R. , Ding, Z.H. *et al*. (2011) A major QTL, Ghd8, plays pleiotropic roles in regulating grain productivity, plant height, and heading date in rice. Mol. Plant, 4, 319–330. 21148627 10.1093/mp/ssq070

[pbi70206-bib-0042] Yang, H.C. , Han, Z.F. , Cao, Y. , Fan, D. , Li, H. , Mo, H.X. , Feng, Y. *et al*. (2012a) A companion cell‐dominant and developmentally regulated H3K4 demethylase controls flowering time in Arabidopsis via the repression of FLC expression. PLoS Genet. 8, 496–512. 10.1371/journal.pgen.1002664 PMC3334889 22536163

[pbi70206-bib-0043] Yang, H.C. , Mo, H.X. , Fan, D. , Cao, Y. , Cui, S.J. and Ma, L.G. (2012b) Overexpression of a histone H3K4 demethylase, JMJ15, accelerates flowering time in Arabidopsis. Plant Cell Rep. 31, 1297–1308. 22555401 10.1007/s00299-012-1249-5

[pbi70206-bib-0044] Yang, Y. , Fu, D.B. , Zhu, C.M. , He, Y.Z. , Zhang, H.J. , Liu, T. , Li, X.H. *et al*. (2015) The RING‐finger ubiquitin ligase HAF1 mediates Heading date 1 degradation during photoperiodic flowering in rice. Plant Cell, 27, 2455–2468. 26296966 10.1105/tpc.15.00320 PMC4815093

[pbi70206-bib-0045] Yano, M. , Katayose, Y. , Ashikari, M. , Yamanouchi, U. , Monna, L. , Fuse, T. , Baba, T. *et al*. (2000) Hd1, a major photoperiod sensitivity quantitative trait locus in rice, is closely related to the arabidopsis flowering time gene CONSTANS. Plant Cell, 12, 2473–2483. 11148291 10.1105/tpc.12.12.2473 PMC102231

[pbi70206-bib-0046] Yin, Y. , Yan, Z. , Guan, J. , Huo, Y. , Wang, T. , Li, T. , Cui, Z. *et al*. (2023) Two interacting basic helix‐loop‐helix transcription factors control flowering time in rice. Plant Physiol. 192, 205–221. 36756926 10.1093/plphys/kiad077 PMC10152653

[pbi70206-bib-0047] Yokoo, T. , Saito, H. , Yoshitake, Y. , Xu, Q. , Asami, T. , Tsukiyama, T. , Teraishi, M. *et al*. (2014) Se14, encoding a JmjC domain‐containing protein, plays key roles in long‐day suppression of rice flowering through the demethylation of H3K4me3 of RFT1. PLoS One, 9, e96064. 24759811 10.1371/journal.pone.0096064 PMC3997562

[pbi70206-bib-0048] Yu, X. , Li, L. , Li, L. , Guo, M. , Chory, J. and Yin, Y. (2008) Modulation of brassinosteroid‐regulated gene expression by Jumonji domain‐containing proteins ELF6 and REF6 in Arabidopsis. Proc. Natl. Acad. Sci. USA, 105, 7618–7623. 18467490 10.1073/pnas.0802254105 PMC2396691

[pbi70206-bib-0049] Yu, Z. , Wang, X. , Wang, Y. , Lu, J. , Chen, H. , Li, X. , Xu, H. *et al*. (2025) Epigenetic regulation of ISPL10 enhances regional adaptability of rice varieties. Plant J. 121, e70109. 40131265 10.1111/tpj.70109

[pbi70206-bib-0050] Zhang, B. , Liu, H.Y. , Qi, F.X. , Zhang, Z.Y. , Li, Q.P. , Han, Z.M. and Xing, Y.Z. (2019a) Genetic interactions among Ghd7, Ghd8, OsPRR37 and Hd1 contribute to large variation in heading date in rice. Rice, 12, 48. 31309345 10.1186/s12284-019-0314-x PMC6629743

[pbi70206-bib-0052] Zhang, S.J. , Hao, H.J. , Liu, X.N. , Li, Y.Y. , Ma, X. , Liu, W.Y. , Zheng, R. *et al*. (2021) SDG712, a putative H3K9‐specific methyltransferase encoding gene, delays flowering through repressing the expression of florigen genes in rice. Rice, 14, 73. 34357443 10.1186/s12284-021-00513-9 PMC8346621

[pbi70206-bib-0051] Zhang, Z.H. , Zhu, Y.J. , Wang, S.L. , Fan, Y.Y. and Zhuang, J.Y. (2019b) Importance of the interaction between heading date genes Hd1 and Ghd7 for controlling yield traits in rice. Int. J. Mol. Sci. 20, 516. 30691093 10.3390/ijms20030516 PMC6387254

[pbi70206-bib-0053] Zhou, S.R. , Cai, L. , Wu, H.Q. , Wang, B.X. , Gu, B. , Cui, S. , Huang, X.L. *et al*. (2024) Fine‐tuning rice heading date through multiplex editing of the regulatory regions of key genes by CRISPR‐Cas9. Plant Biotechnol. J. 22, 751–758. 37932934 10.1111/pbi.14221 PMC10893950

[pbi70206-bib-0054] Zong, W.B. , Ren, D. , Huang, M.H. , Sun, K.L. , Feng, J.L. , Zhao, J. , Xiao, D.D. *et al*. (2021) Strong photoperiod sensitivity is controlled by cooperation and competition among Hd1, Ghd7 and DTH8 in rice heading. New Phytol. 229, 1635–1649. 33089895 10.1111/nph.16946 PMC7821112

