# Supplementary material for: JMJ720 regulates flowering time in rice via H3K9 demethylation of Hd1
Source: Plant Biotechnol J. 2025 Jun 15;23(9):3824–37. doi: 10.1111/pbi.70206 (PMC12392927; doi:10.1111/pbi.70206)
Supplement: Supplementary file 1 — Figure S1 Identification of JMJ720. Figure S2 The jmj720 mutant in the ZH11 background accelerates rice flowering. Figure S3 The jmj720 mutants exhibited no significant differences in agronomic traits compared to TH899. Figure S4 JMJ720 regulates flowering‐related gene expression. Figure S5 JMJ720 OE does not change the flowering in the ZH11 background. Figure S6 JMJ720 does not interact with Hd1. Figure S7 Characterization of POH OE plants in ZH11 background and jmj720‐3 mutant. Figure S8 ChIP assays showing that the enrichment of Hd1 in POH1 OE plants. Figure S9 Characterization of poh1, hd1 jmj720 and hd1 jmj720 poh1 mutants in ZH11 background. Figure S10 Association analysis of JMJ720 haplotypes with flowering time in rice germplasm from RiceAtlas database. [file PBI-23-3824-s002.docx]

**Supplemental Figure 1. Identification of JMJ720*.***

(A) Distribution of SNPs by BSA-seq and locations of heading date genes on rice chromosomes.

(B) Expression analysis of *JMJ720* in TH899, K4 and complemented plants.

**Supplemental Figure 2. The *jmj720* mutant in the ZH11 background accelerates rice flowering.**

(A) Schematic map of the genomic region of *JMJ720* and the sgRNA target site. The PAM motif (NGG) is shown in red.

(B) Sequencing chromatograms of *jmj720* in TH899, triangle means deletion and square means replace as indicated.

(C) Sequencing chromatograms of *jmj720* in ZH11, triangle means deletion or insertion as indicated.

(D) Phenotype of *jmj720* mutants and ZH11 grown under LD conditions.
(E) Heading date of *jmj720* mutants and ZH11 grown under LD and SD conditions. Data are means ± standard error (SE; *n* = 10). Statistically significant differences are indicated by different lowercase letters (*P* < 0.05, one-way ANOVA with Tukey’s significant difference test).

**Supplemental Figure 3. The *jmj720* mutants exhibited no significant differences in agronomic traits compared to TH899.**

(A-F) Statistical analysis of plant height (A), panicle number (B), panicle length (C), grain number per panicle (D), 1000 grain weight (E), yield per plant (F) between the *jmj720* mutants and TH899 for two consecutive years. Data are means ± standard error (SE; *n* = 10). ns means no significance.

**Supplemental Figure 4. JMJ720 regulates flowering-related gene expression.**
(A, C, E) Rhythmic expression patterns of *Hd2* (A)*, Hd4* (C), and *Hd5* (E) in *jmj720* and TH899 plants under LD conditions.

(B, D, F) Rhythmic expression patterns of *Hd2* (B)*, Hd4* (D), and *Hd5* (F) in *jmj720* and TH899 plants under SD conditions.

Black and white boxes denote dark and light periods, respectively. *UBIQUITIN* was used as the internal control. Data are means ± SE (*n* = 3). ZT, zeitgeber time.

**Supplemental Figure 5. *JMJ720 OE* does not change the flowering in the ZH11 background.**

(A) Phenotype of *JMJ720 OE* plants and ZH11 grown under LD conditions.

(B) Protein gel blot showing *JMJ720* is overexpressed in *JMJ720 OE* lines. ZH11 was used as control. The immunoblot was probed with anti-flag antibody. Actin contents detected with anti-Actin antibody were used as loading control.

(C) RT-qPCR analysis of *JMJ720* transcription level in *JMJ720 OE* lines and ZH11. The expression level in ZH11 was set as “1”, data was shown as means ± SE (*n*=3).
(D) Heading date of *JMJ720 OE* plants and ZH11 under LD and SD conditions. Data are means ± standard error (SE; *n* = 10). *P* values were calculated by Student’s *t* test compared to ZH11.

**Supplemental Figure 6. JMJ720 does not interact with Hd1.**

(A) JMJ720 could not interact with Hd1 in yeast cells. The indicated construct pairs were cotransformed into yeast strain Y2H.

(B) LUC complementation assay shows JMJ720 could not interact with Hd1 in *N. benthamiana* leaves.

**Supplemental Figure 7. Characterization of *POH OE* plants in ZH11 background and *jmj720-3* mutant*.***

(A) Phenotype of *POH1 OE* plants and ZH11 grown under SD conditions.

(B) RT-qPCR analysis of *POH1* transcription level in *POH1 OE*, *jmj720-3 POH1 OE* lines and ZH11. The expression level in ZH11 was set as “1”, data was shown as means ± SE (*n*=3).

**Supplemental Figure 8. ChIP assays showing that the enrichment of POH1 at *Hd1* promoter.**

Immunoprecipitation was performed with anti-Myc antibody in ZH11 and *POH1 OE*. The enrichment at *Hd1* was quantified by qPCR. Data was shown as means ± SE (*n*=3) relative to ZH11. *P* values were calculated by Student’s *t* test, ** is *P* < 0.01.

**Supplemental Figure 9. Characterization of *poh1, hd1 jmj720*, and *hd1 jmj720 poh1* mutants in ZH11 background*.***

(A) Sequencing chromatograms of *hd1 jmj720*, *hd1 jmj720 poh1* in ZH11, triangle means deletion or insertion as indicated.

(B) Sequencing chromatograms of *poh1* in ZH11, triangle means deletion or insertion as indicated.

(C) Phenotype of *poh1* mutants and ZH11 grown under SD conditions.
(D) Heading date of *poh1* mutants and ZH11 grown under SD conditions. Data are means ± standard error (SE; *n* = 10). *P* values were calculated by Student’s *t* test compared to ZH11.

**Supplemental Figure 10. Association analysis of JMJ720 haplotypes with flowering time in rice germplasm from RiceAtlas database.**
